# Supplementary material for: Negative Regulation of the Differentiation of Flk2− CD34− LSK Hematopoietic Stem Cells by EKLF/KLF1
Source: Int J Mol Sci. 2020 Nov 10;21(22):8448. doi: 10.3390/ijms21228448 (PMC7697791; doi:10.3390/ijms21228448)
Supplement: Supplementary file 1 [file ijms-21-08448-s001.pdf]

Fig.1

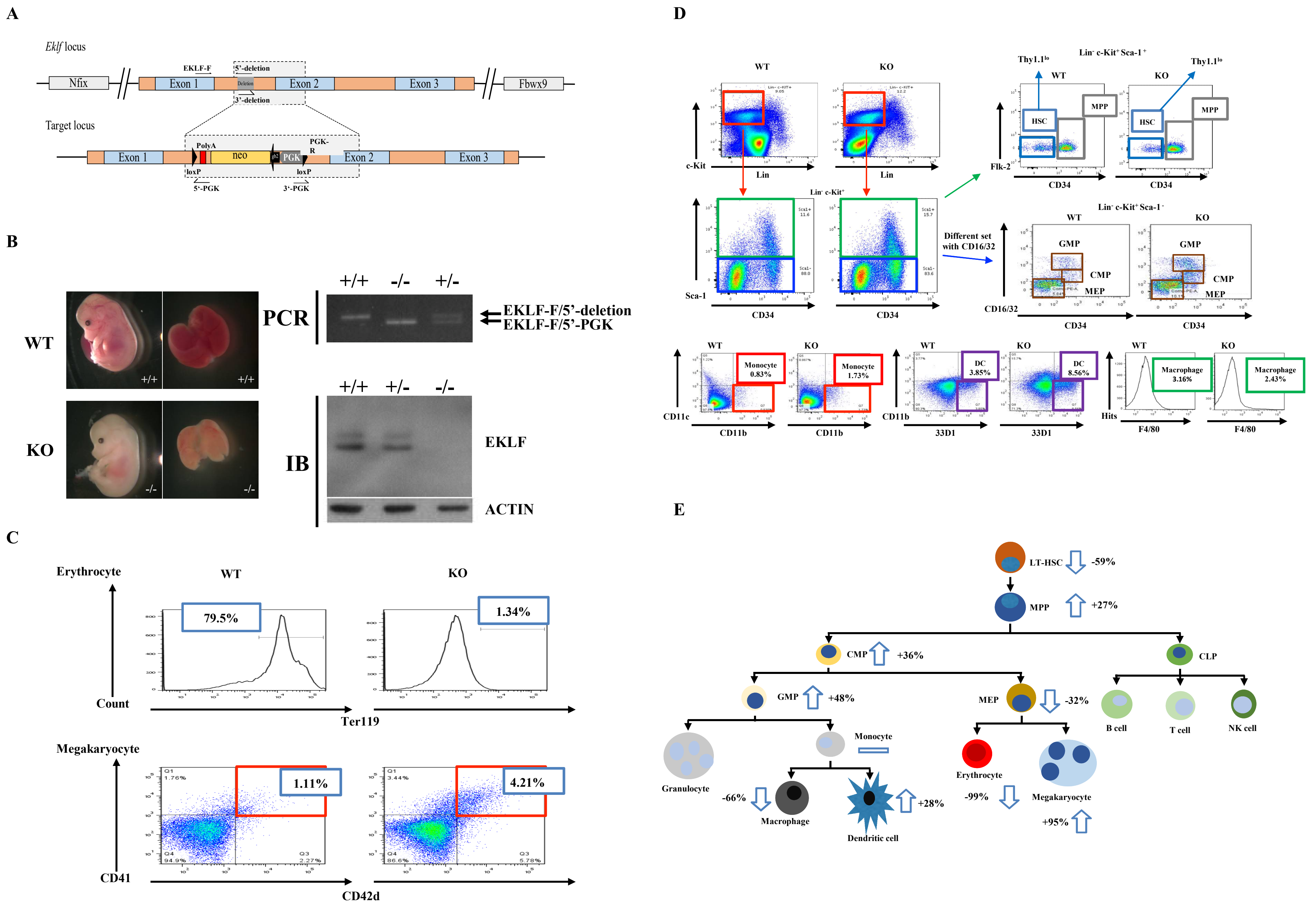

A
Eklf locus
Nfix
EKLF-F
5'-deletion
Exon 1
Exon 2
Exon 3
3'-deletion
Fbwx9
Target locus
PolyA
neo
PGK
PGK-R
loxP
5'-PGK
3'-PGK
B
WT
KO
+/+
-/-
PCR
+/+ -/- +/-
EKLF-F/5'-deletion
EKLF-F/5'-PGK
IB
+/+ +/- -/-
EKLF
ACTIN
C
Erythrocyte
WT
KO
79.5%
1.34%
Count
Ter119
Megakaryocyte
1.11%
4.21%
CD41
CD42d
D
WT
KO
c-Kit
Lin
Lin- c-Kit+
Sca-1
CD34
Lin- c-Kit+ Sca-1+
Thy1.1lo
HSC
MPP
Flk-2
Different set with CD16/32
Lin- c-Kit+ Sca-1-
GMP
CMP
MEP
CD16/32
Monocyte 0.83%
Monocyte 1.73%
CD11c
CD11b
DC 3.85%
DC 8.56%
33D1
Macrophage 3.16%
Macrophage 2.43%
Hits
F4/80
E
LT-HSC -59%
MPP +27%
CMP +36%
CLP
GMP +48%
MEP -32%
B cell
T cell
NK cell
Granulocyte
Monocyte
Erythrocyte
Megakaryocyte
-66%
Macrophage
Dendritic cell
+28%
-99%
+95%

Fig.2

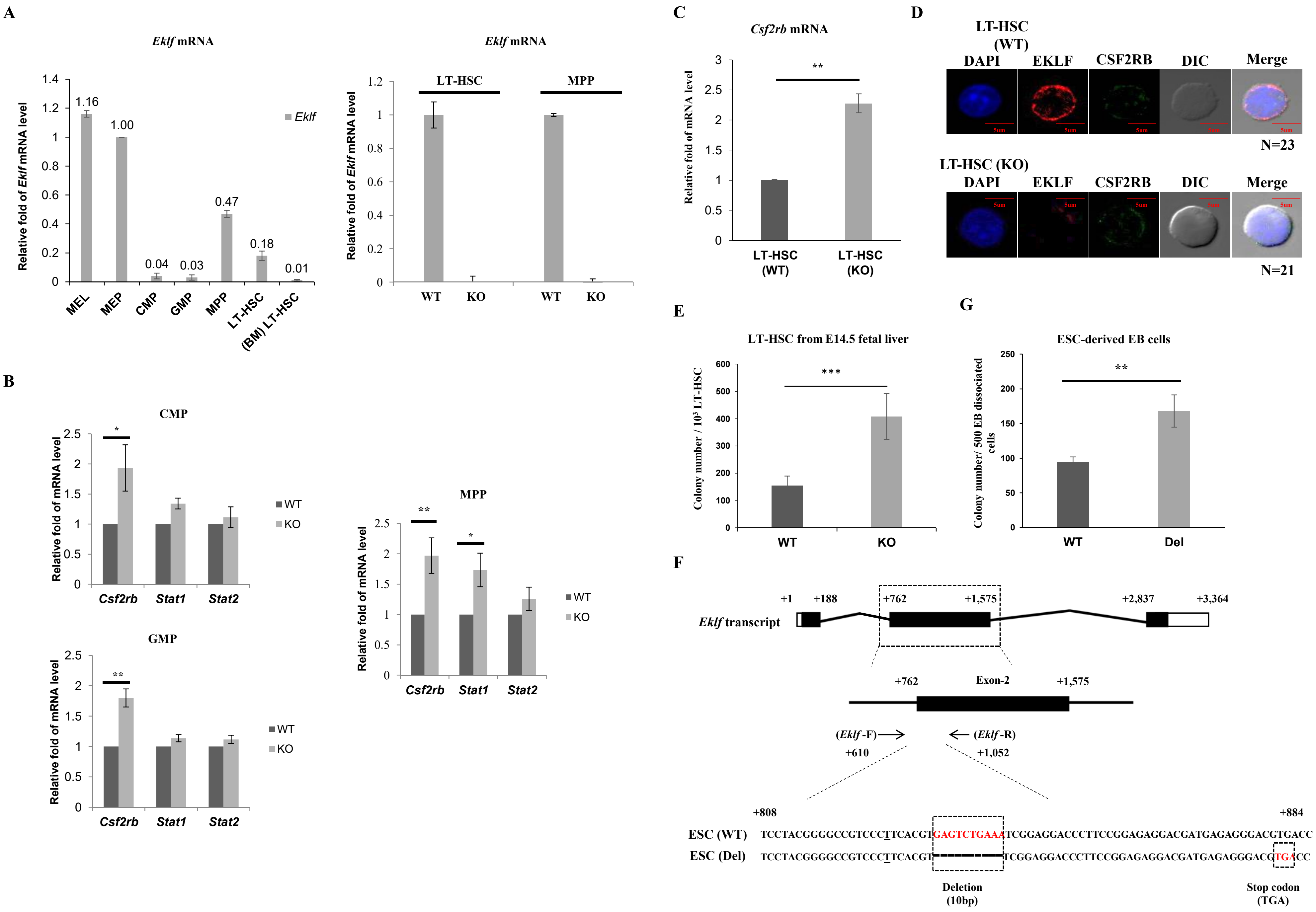
A
Eklf mRNA
Relative fold of Eklf mRNA level
Eklf
1.16
1.00
0.04
0.03
0.47
0.18
0.01
MEL
MEP
CMP
GMP
MPP
LT-HSC
(BM) LT-HSC
Eklf mRNA
Relative fold of Eklf mRNA level
LT-HSC
MPP
WT
KO
WT
KO
B
CMP
Relative fold of mRNA level
*
WT
KO
Csf2rb
Stat1
Stat2
GMP
Relative fold of mRNA level
**
WT
KO
Csf2rb
Stat1
Stat2
MPP
Relative fold of mRNA level
**
*
WT
KO
Csf2rb
Stat1
Stat2
C
Csf2rb mRNA
Relative fold of mRNA level
**
LT-HSC (WT)
LT-HSC (KO)
D
LT-HSC (WT)
DAPI
EKLF
CSF2RB
DIC
Merge
N=23
LT-HSC (KO)
DAPI
EKLF
CSF2RB
DIC
Merge
N=21
E
LT-HSC from E14.5 fetal liver
Colony number / 10³ LT-HSC
***
WT
KO
G
ESC-derived EB cells
Colony number/ 500 EB dissociated cells
**
WT
Del
F
+1
+188
+762
+1,575
+2,837
+3,364
Eklf transcript
+762
Exon-2
+1,575
(Eklf-F)
(Eklf-R)
+610
+1,052
+808
+884
ESC (WT) TCCTACGGGGCCGTCCCTTCACGTGAGTCTGAAATCGGAGGACCCTTCCGGAGAGGACGATGAGAGGGACGTGACC
ESC (Del) TCCTACGGGGCCGTCCCTTCACGT----------TCGGAGGACCCTTCCGGAGAGGACGATGAGAGGGACCTGACC
Deletion (10bp)
Stop codon (TGA)

Fig.3

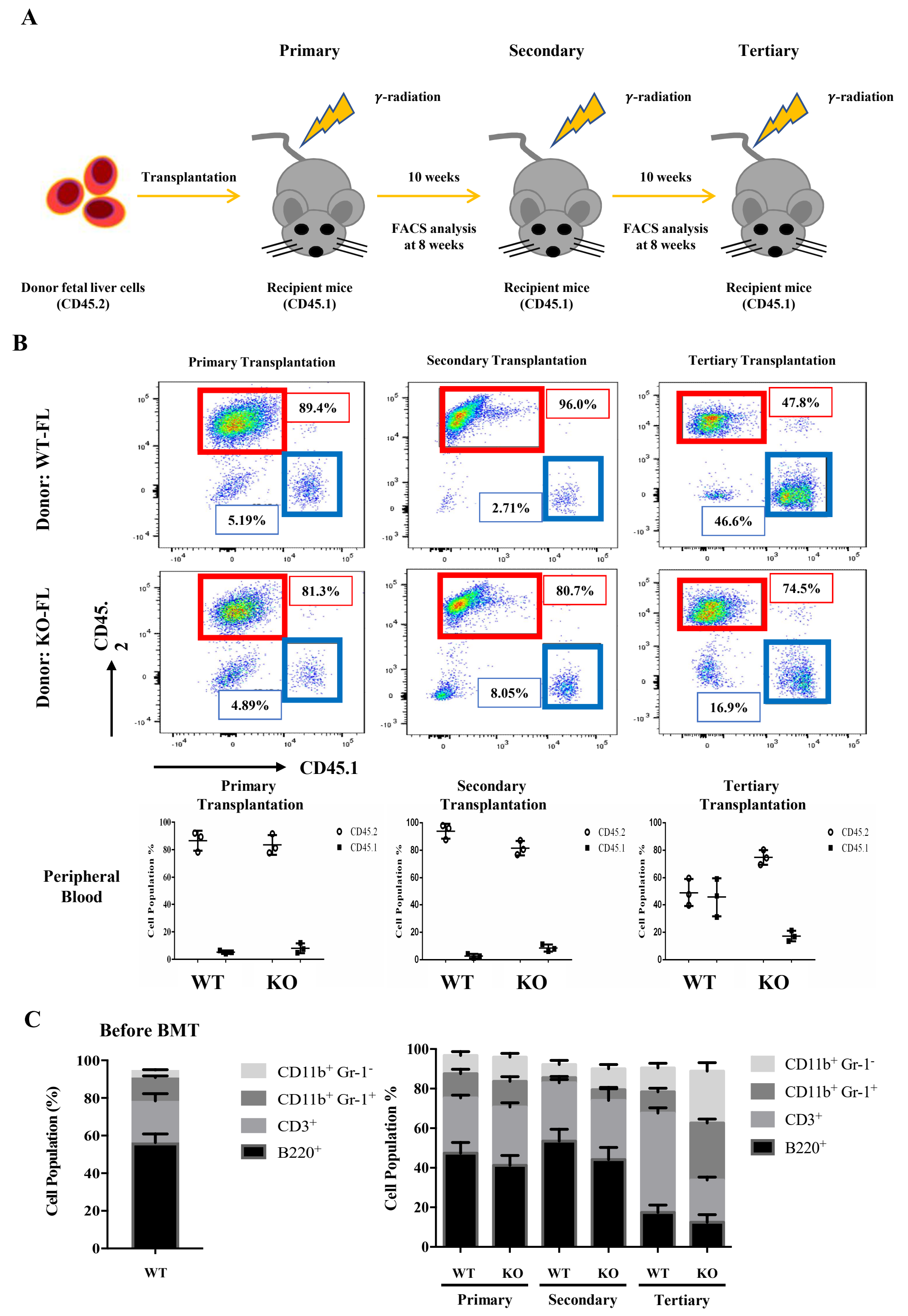
A
Primary
Secondary
Tertiary
γ-radiation
Transplantation
10 weeks
FACS analysis at 8 weeks
Donor fetal liver cells (CD45.2)
Recipient mice (CD45.1)
B
Primary Transplantation
Secondary Transplantation
Tertiary Transplantation
Donor: WT-FL
Donor: KO-FL
89.4%
5.19%
96.0%
2.71%
47.8%
46.6%
81.3%
4.89%
80.7%
8.05%
74.5%
16.9%
CD45.2
CD45.1
Peripheral Blood
Cell Population %
CD45.2
CD45.1
WT
KO
C
Before BMT
Cell Population (%)
CD11b+ Gr-1-
CD11b+ Gr-1+
CD3+
B220+
Cell Population %
Primary
Secondary
Tertiary

Fig.4

A

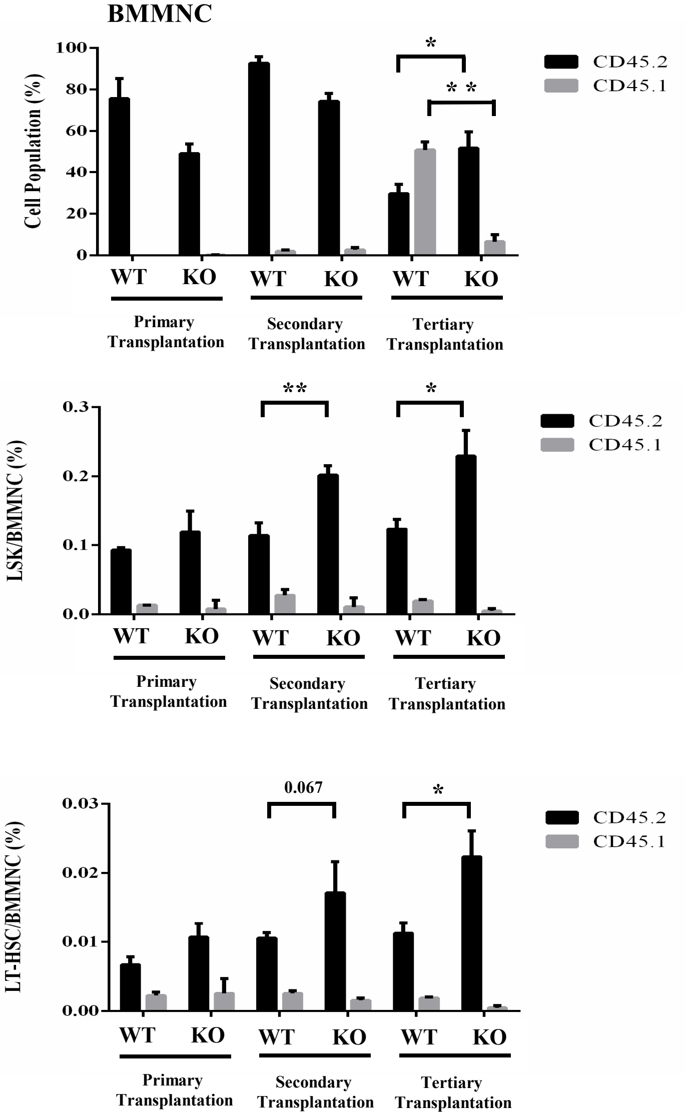
BMMNC
Cell Population (%)
100
80
60
40
20
0
CD45.2
CD45.1
*
* *
WT
KO
WT
KO
WT
KO
Primary Transplantation
Secondary Transplantation
Tertiary Transplantation
LSK/BMMNC (%)
0.3
0.2
0.1
0.0
**
*
CD45.2
CD45.1
WT
KO
WT
KO
WT
KO
Primary Transplantation
Secondary Transplantation
Tertiary Transplantation
LT-HSC/BMMNC (%)
0.03
0.02
0.01
0.00
0.067
*
CD45.2
CD45.1
WT
KO
WT
KO
WT
KO
Primary Transplantation
Secondary Transplantation
Tertiary Transplantation

B

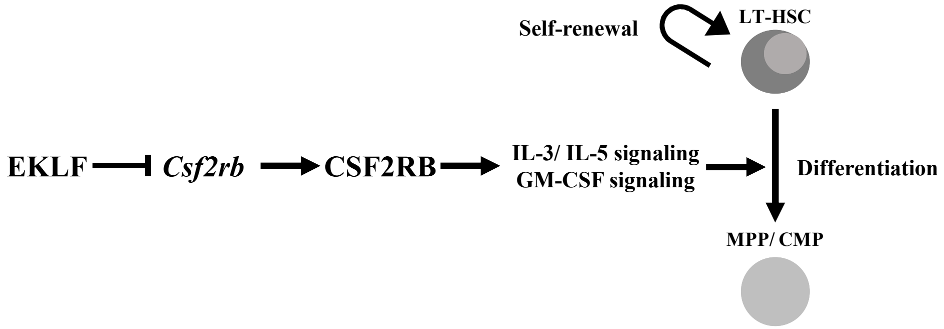
Self-renewal
LT-HSC
EKLF
Csf2rb
CSF2RB
IL-3/ IL-5 signaling
GM-CSF signaling
Differentiation
MPP/ CMP

Supplementary Table 1. Number/ fetal liver of different types of hematopoietic cells in E14.5 fetal livers from WT and *Eklf* $^{-/-}$ embryos, respectively

	Cell number x10^6 / E14.5 Fetal Liver	
**Cell Types**	WT	KO
**Erythrocyte**	23.4±0.02	0.2±0.03 (-99%)
	**Cell number x10^3 / E14.5 Fetal Liver**	
**Cell Types**	WT	KO
**LT-HSC**	7.1±1.2	2.9±0.3 (-59%)
**MPP**	19.0±3.5	24.3±2.0 (+27%)
**LSK**	26.1±3.2	27.2±3.3 (+4%)
	**Cell number x10^4 / E14.5 Fetal Liver**	
**CMP**	14.7±2.7	19.9±2.9 (+36%)
**GMP**	26.6±6.3	39.6±7.7 (+48%)
**MEP**	31.9±4.4	21.5±1.5 (-32%)
	**Cell number x10^5 / E14.5 Fetal Liver**	
**Monocyte**	6.8±0.7	6.9±1.0 (+1.5%)
**Macrophage**	7.7±1.7	2.6±1.2 (-66%)
**Megakaryocyte**	32.9±2.3	64.4±5.4 (+95%)
**Dendritic cell**	15.2±2.0	19.6±0.9 (+28%)

The E14.5 fetal liver cells were analyzed by FACS, and the average cell number/ fetal liver of each cell types is presented. Differences of the cell numbers of the KO fetal liver relative to the WT fetal liver are shown in the parentheses as percentages (%) which are also shown in Fig 1E. N≥6

Supplementary Table 2. List of surface markers used for detection and/or sorting of different types of hematopoietic cells from fetal liver, bone marrow or peripheral blood

Cell type	Surface Markers
LT-HSC	Lin^- $c\text{-}Kit^+$ $Sca\text{-}1^+$ ($Thy1.1^{lo}$) $Flk2^-$ $CD34^-$
BM LT-HSC	Lin^- $c\text{-}Kit^+$ $Sca\text{-}1^+$ $Flk2^-$ $CD34^-$
MPP	Lin^- $c\text{-}Kit^+$ $Sca\text{-}1^+$ ($Thy1.1^-$) $Flk2^+$ $CD34^+$
CMP	Lin^- $c\text{-}Kit^+$ $Sca\text{-}1^-$ $CD34^+$ $CD16/32^{int}$
GMP	Lin^- $c\text{-}Kit^+$ $Sca\text{-}1^-$ $CD34^+$ $CD16/32^{hi}$
MEP	Lin^- $c\text{-}Kit^+$ $Sca\text{-}1^-$ $CD34^-$ $CD16/32^{lo/int}$
Monocyte	$CD11b^+$ $CD11c^-$ or $CD11b^+$ $Gr\text{-}1^-$
Macrophage	$F4/80^+$
Dendritic cells	$CD11b^-$ $33D1^+$
Erythrocyte	$Ter119^+$
Megakaryocyte	$CD41^+$ $CD42d^+$
Granulocyte	$CD11b^{hi}$ $Gr\text{-}1^{hi}$ or $CD11b^+$ $Gr\text{-}1^+$
T cells	$CD3^+$
B cells	$B220^+$

Supplementary Table 3. List of PCR primers used in RT-qPCR analysis of the mRNAs and sequences of the DNA oligos used for CRISPR/Cas9 editing of ES cells

	Forward	Reverse
Eklf	5'-GGACACCCAGGAGGACTTC-3'	5'-GGGTCCTCCGATTTCAGACTCA-3'
Actin	5'-ATGGAGGGGAATACAGCCC-3'	5'-TTCTTTGCAGCTCCTTCGT-3'
Csf2rb	5'-ACAGAGAACCTAGATCGAGCC-3'	5'-GTGTACTCTTCGCTCCACTTG-3'
Stat1	5'-CTGAATATTTCCCTCCTGGG -3'	5'-TCCCGTACAGATGTCCATGAT-3'
Stat2	5'-GCTGTCAAGGTTCTGCAACA-3'	5'-CGCTTGGAGAATTGGAAGTT-3'
Fcer1a	5'-GAGTGCCACCGTTCAAGACA-3'	5'-GTAGATCACCTTGCGGACATTC-3'

Name	Sequence	Applications
Eklf-gRNA-F	5'-acctTCACGTGAGTCTGAAATCGG-3'	Construction of the *Eklf*-gRNA expression plasmid
Eklf-gRNA-R	5'-aaacCCGATTTCAGACTCACGTGA-3'	
Eklf-F	5'-CTAGCAAGACCTAATGGAGTTGTC-3'	PCR across the Cas9/gRNA target site of *Eklf*
Eklf-R	5'-CAAAGGCCCAGTCACCAACC-3'	

F-forward primer/ R-reverse primer
